# Serological evidence of sarbecovirus exposure along Sunda pangolin trafficking pathways

**DOI:** 10.1186/s12915-024-02074-x

**Published:** 2024-11-26

**Authors:** Brian M. Worthington, Portia Y.-H. Wong, Kishoree K. Kumaree, Tracey-Leigh Prigge, Kar Hon Ng, Yunshi Liao, Paolo Martelli, Sarah Churgin, Foo K. Lee, Chris Perkins, Michael Bradley, Mac P. Pierce, Marcus H.-H. Shum, Elliott F. Miot, William Y.-M. Cheung, Shelby E. McIlroy, Helen C. Nash, Wirdateti, Gono Semiadi, Chee-Wah Tan, Lin-Fa Wang, Gary Ades, David M. Baker, Caroline Dingle, Oliver G. Pybus, Edward C. Holmes, Gabriel M. Leung, Yi Guan, Huachen Zhu, Timothy C. Bonebrake, Tommy T. Y. Lam

**Affiliations:** 1 State Key Laboratory of Emerging Infectious Diseases, School of Public Health, The University of Hong Kong, Hong Kong SAR, People’s Republic of China; 2 Guangdong-Hongkong Joint Laboratory of Emerging Infectious Diseases, Joint Institute of Virology (Shantou University, The University of Hong Kong), Shantou, Guangdong 515063 People’s Republic of China; 3 Advanced Pathogen Research Institute, Futian District, Shenzhen City, Guangdong, 518045 People’s Republic of China; 4 Centre for Immunology & Infection Limited, Hong Kong SAR, People’s Republic of China; 5 School of Biological Sciences, The University of Hong Kong, Hong Kong SAR, People’s Republic of China; 6 Marine and Environmental Biology Section, Department of Biology, University of Southern California, Los Angeles, CA USA; 7 Ocean Park Corporation, Hong Kong SAR, People’s Republic of China; 8 Ocean Park Conservation Foundation, Hong Kong SAR, People’s Republic of China; 9 Laboratory of Data Discovery for Health Limited, Hong Kong SAR, People’s Republic of China; 10 HKU-Pasteur Research Pole, The University of Hong Kong, Hong Kong SAR, People’s Republic of China; 11 MIVEGEC, Université de Montpellier, IRD, CNRS, 34394 Montpellier, France; 12 School of Life Sciences, Simon F.S. Li Marine Science Laboratories, The Chinese University of Hong Kong, Shatin, Hong Kong SAR, People’s Republic of China; 13 Department of Biological Sciences, National University of Singapore, 14 Science Drive 4, Singapore, Singapore; 14 Research Centre for Ecology and Innovation Agency, BRIN, Cibinong, 16911 Indonesia; 15 Research Centre for Biosystematics and Evolution, BRIN, Cibinong, 16911 Indonesia; 16 Infectious Diseases Translational Research Programme, Department of Microbiology and Immunology, Yong Loo Lin School of Medicine, National University of Singapore, Singapore, Singapore; 17 Programme in Emerging Infectious Diseases, Duke–National University of Singapore Medical School, Singapore, Singapore; 18 Kadoorie Farm and Botanic Garden, Lam Kam Road, Tai Po, Hong Kong SAR, People’s Republic of China; 19 School of Biological Sciences, Swire Institute of Marine Science, The University of Hong Kong, Hong Kong SAR, People’s Republic of China; 20 Department of Biology, University of Oxford, Oxford, UK; 21 Department of Pathobiology and Population Sciences, The Royal Veterinary College, London, UK; 22 Sydney Institute for Infectious Diseases, School of Medical Sciences, University of Sydney, Sydney, NSW 2006 Australia; 23 Shanghai Institute of Virology, Shanghai Jiao Tong University School of Medicine, 227 South Chongqing Road, Shanghai, 200225 People’s Republic of China

**Keywords:** One Health, Pangolins, SARS-related virus, Sarbecovirus, Coronavirus, Paramyxovirus, Population genomics, Serology, ELISA, Conservation forensics

## Abstract

**Background:**

Early in the coronavirus disease 2019 (COVID-19) pandemic, Sunda pangolins (*Manis javanica*) involved in the illegal wildlife trade in mainland China were identified as hosts of severe acute respiratory syndrome-related coronaviruses (SARSr-CoVs). Although it is unconfirmed whether pangolins or other traded wildlife served as intermediate hosts for severe acute respiratory syndrome coronavirus 2 (SARS-CoV-2), the trafficking of pangolins presents a clear risk for transmission of viruses with zoonotic and epizootic potential regardless. We have investigated the origins of pangolin carcasses seized in Hong Kong and have evaluated their potential exposure to SARSr-CoVs, other coronaviruses, and paramyxoviruses, aiming to address a gap in our knowledge with regard to the role of wildlife trade in the maintenance and emergence of pathogens with zoonotic and epizootic potential.

**Results:**

Using a combination of virological and wildlife forensics tools, we investigated 89 Sunda pangolin carcasses seized by Hong Kong authorities during anti-smuggling operations in the territory conducted in 2013 (*n* = 1) and 2018 (*n* = 88). Swabs, organ tissues, blood, and other body fluids were collected during post-mortem examination. Two enzyme-linked immunosorbent assays (ELISAs), which employ a double-antigen sandwich format, were used to detect antibodies reactive against SARSr-CoVs. One individual was found to be seropositive with support from both methods, while five individuals exhibited a putatively seropositive result from one ELISA method. Polymerase chain reaction (PCR) screening for coronavirus and paramyxovirus ribonucleic acid (RNA) did not yield any positives. Based on genomic data, the seropositive individual was determined to have likely originated from Java, while the putatively seropositive individuals were determined to have originated from populations in Borneo, Java, and Singapore/Sumatra.

**Conclusions:**

While the role of pangolins in the evolution and ecology of SARS-CoV-2 is uncertain, our results suggest susceptibility and potential exposure of pangolins to SARSr-CoVs, occurring naturally or associated with the illegal trafficking of these animals. Complex dynamics between natural populations, traded individuals, and pathogen susceptibility complicate conclusions about the role of pangolins, as well as other host species, in the ecology of SARSr-CoVs and potentially zoonotic viruses with risk of future emergence.

**Supplementary Information:**

The online version contains supplementary material available at 10.1186/s12915-024-02074-x.

## Background

The discovery of novel SARSr-CoVs bearing high similarity to SARS-CoV-2 in trafficked Sunda pangolins (*Manis javanica*) in late 2019 and early 2020 propelled the global illegal trade of pangolins into the public spotlight [1–3]. While it is known that the transnational trade of wildlife products poses risks for pathogen introduction and zoonotic disease transmission [4, 5], the precise relationship between wildlife trade and the COVID-19 pandemic remains unclear. During the SARS outbreak, a SARS-like virus was identified in palm civets and other small mammals sold at markets in southern China [6]. Subsequent efforts have identified diverse viruses of the genus *Sarbecovirus* in pangolins and other species such as horse-shoe (i.e., genus *Rhinolophus*) bats [1–3, 7–15]. While the majority of sarbecoviruses have been characterized from bats, the discovery of diverse SARSr-CoVs in trafficked pangolins indicates that pangolins are susceptible to infection and have the potential to play a role in the transmission dynamics of SARSr-CoVs, presenting a clear risk for human exposure through the exploitation of these animals.

Of the eight currently recognized pangolin species, all are listed as threatened and three as critically endangered (*Manis javanica* [16]; *Manis pentadactyla* [17]; *Manis culionensis* [18]). Despite restrictions on international trade, demand for pangolin products has persisted, such that pangolins are considered the most trafficked mammal in the world, with the Sunda pangolin being the most commonly traded Asian species [19–21]. High levels of illegal international wildlife trade can threaten biodiversity and ecosystems involving pangolins by causing population declines, especially of vulnerable or threatened species, which ultimately lead to ecosystem-level impacts through extirpation [22, 23].

Beyond continued exploitation of already threatened populations by ongoing illegal trade, pangolin trafficking further exacerbates zoonotic and epizootic disease risks, presenting significant challenges for global pathogen surveillance and monitoring [24, 25]. Exploitation of pangolins and other wildlife through trade increases human exposure to potential reservoirs of zoonotic pathogens by expanding the interface between humans, domestic animals, and wildlife. This creates opportunities for cross-species transmission within trade networks, both at upstream sites closer to wildlife origins and at downstream interfaces closer to consumers. Changing interface dynamics between host species may contribute to the maintenance and emergence of zoonotic or epizootic pathogens in novel hosts [26, 27]. The poor welfare conditions experienced by trafficked wildlife, including confinement in unhygienic conditions, overcrowding, and increased contact with other species, compound infectious disease risks. Such factors may impair their resistance to infection while increasing their exposure to diverse pathogens, effectively lowering the barrier for cross-species transmission and pathogen emergence [28].

Given the public health threats prompted by the illegal wildlife trade and the discovery of multiple SARSr-CoVs in pangolins, it is crucial to investigate the relationship between pangolins and SARSr-CoVs to identify potential spillover risks. At present, however, the Sunda pangolin trade network is still largely unknown due to its complexity and illicit nature, lack of documentation, and the incomprehensive nature of seizure reports, complicating efforts to combat the trade and detect the origins of trafficked animals and their associated pathogens [29–31]. While previous efforts have been undertaken to map the Sunda pangolin trade (e.g., Nash et al. [32] and Hu et al. [33]) or to identify various viruses of zoonotic or epizootic concern found in pangolins (e.g., Chen et al. [34], Cui et al. [35], Gao et al. [36], Liu et al. [2], Nga et al. [11], Shi et al. [37]), studies that simultaneously investigate the origins of traded pangolins and characterize their associated pathogens are lacking, despite the importance of understanding these associations to mitigate the risk of potential spillover events.

In this study, we investigated the origins of 89 Sunda pangolin carcasses seized by law enforcement while being trafficked through Hong Kong and examined their potential exposure to SARSr-CoVs. Using population genomics methods (sensu Nash et al. [32]), we assigned pangolin individuals to one of three regions within their geographic range (Java, Borneo, and Singapore/Sumatra), while simultaneously screening for coronaviruses and paramyxoviruses in samples collected during post-mortem examination. Additionally, serological methods were employed for the detection of antibodies with cross-reactivity to SARSr-CoVs. Through this integrated approach, we explored possible routes of natural exposure of pangolins to SARSr-CoVs, as well as the role of wildlife trade in the maintenance and emergence of viruses with zoonotic and epizootic potential.

## Results

### Necropsy examination highlights similarity in slaughter methods used across regions

A total of 40 male and 49 female Sunda pangolin (*Manis javanica*) carcasses were investigated as part of this study. In all cases, there was apparent ante-mortem cranial bruising, with evidence that slaughter occurred by means of a transverse incision across the ventral neck, cutting deep and sectioning all structures through to the cervical spine. Various wounds and bruising were also observed on extremities, suspected to be inflicted through the use of snares as well as potential dog-inflicted wounds.

All carcasses had been denuded and eviscerated prior to packing, with evidence of having been scalded in boiling water to facilitate the removal of scales and claws prior to dressing of the carcasses. The abdominal cavity was opened ventrally by an incision from the pelvis to the lower abdomen. All major respiratory, digestive, and reproductive organs, including fetuses in various stages of development, had been removed from most individuals. Organs had been rinsed, transferred to a plastic bag, and returned to the abdominal cavity. Red hepatization with gray and black discoloration was apparent on the lungs in most individuals where lungs were present and crater-like ulcers and lesions were frequently observed on the interior stomach lining.

### Population genomics reveal three origins of trafficked pangolins

**Fig. 1:**
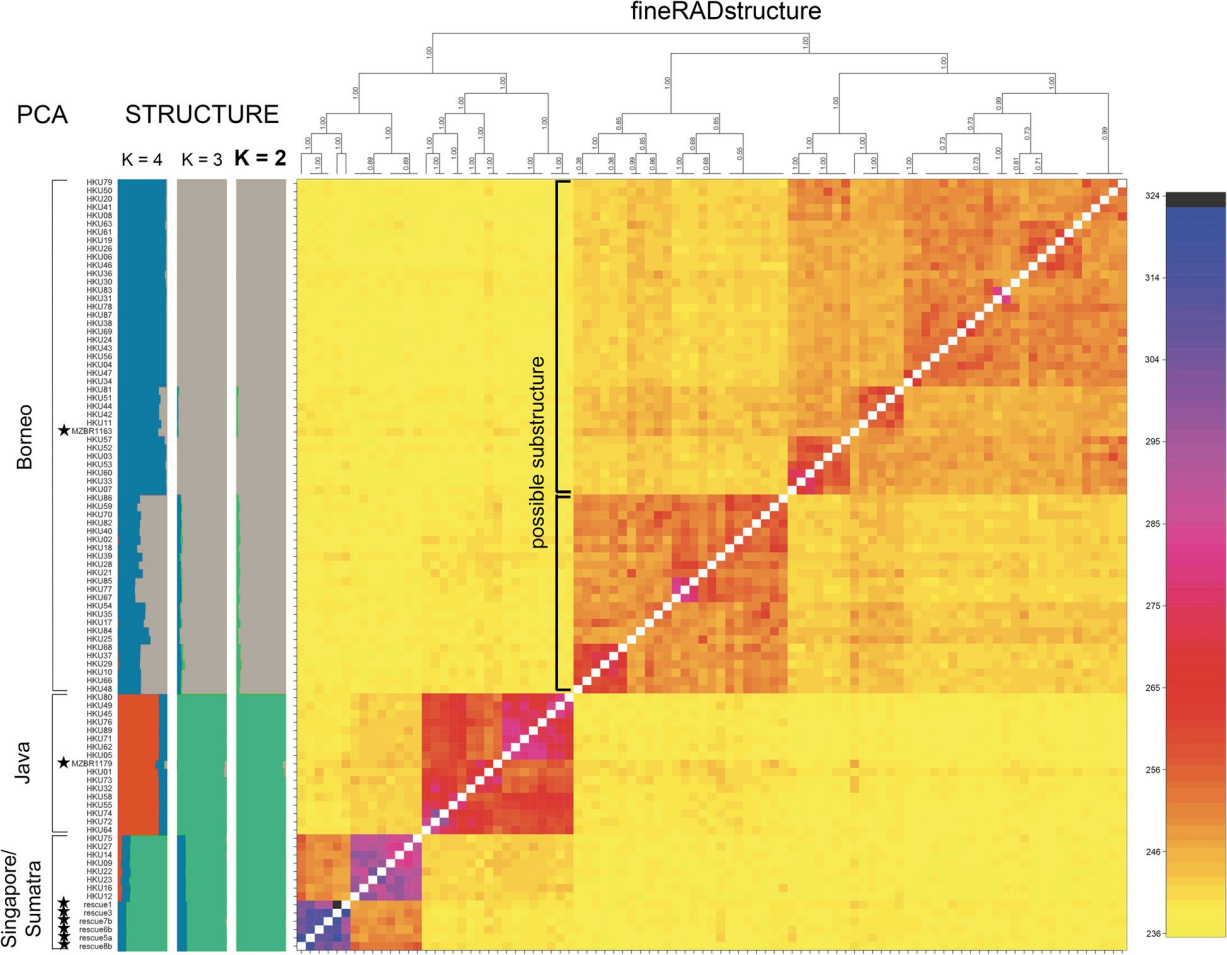
Three geographic origins of trafficked Sunda pangolins shown by the principal components analysis, STRUCTURE analysis, and fineRADstructure analyses. Analyses were based on 23,261 SNPs of 85 Sunda pangolin individuals from this study and 8 references. Color of each grid from the fineRADstructure plot corresponds to the color scale on the right, where grids towards the purple end of the spectrum show higher estimated coancestry between the two individuals. Individuals marked with a star are references from Nash et al. [32]. Possible subclusters within Borneo are marked with brackets within the fineRADstructure plot

To assign population origin of the seized pangolins, we used double digest restriction site-associated deoxyribonucleic acid (DNA) sequencing (ddRADseq) to identify genome-wide single nucleotide polymorphisms (SNPs) in all 89 seized individuals and attribute these to one of three populations identified by Nash et al. [32] and Sitam et al. [38]. We initially obtained 37,775 genome-wide SNPs from ref_map.pl from 101 individuals (89 from this study and 12 references from Nash et al. [32]; Additional file 2). Through the mind program on PLINK, 8 individuals (4 samples and 4 references; Additional file 2) were filtered out for missing data, while 14,514 SNPs were filtered out using the maf, hwe, and indep-pairwise programs. BayeScan analysis found no sign of selection in the SNPs. Ultimately, we retained 23,261 SNPs from 93 individuals (85 from this study and 8 references) for downstream analysis. From the principal component analysis (PCA), the 85 Sunda pangolin individuals from this study originate from three different geographic regions: Borneo (*n* = 61), Java (*n* = 16), and Singapore/Sumatra (*n* = 8) (Fig. 1 and Additional file 1). The results from STRUCTURE differed slightly, with *K* = 2 as the most likely scenario following the Evanno method [39], although these differences were resolved through the haplotype-based analysis in fineRADstructure, ultimately showing the three origin regions. The fineRADstructure results also suggest that the cluster from Borneo should be divided into two groups (Fig. 1), indicating the presence of some population substructure within the Borneo population. Given the geographic limitation of the populations identified by Nash et al. [32], patterns of population structure inferred from this data are also limited to the southern island region of the range of Sunda pangolins.

### Virological investigation unable to detect coronavirus and paramyxovirus RNA

Swab and tissue samples collected from the 89 seized pangolin carcasses were screened for the presence of coronavirus and paramyxovirus RNA. Conventional reverse transcription PCR (RT-PCR) for detection of coronaviruses and paramyxoviruses was performed for 815 swab and tissue samples. No viral RNA was detected. Additionally, specific detection of sarbecoviruses by reverse transcription-quantitative PCR (RT-qPCR) using consensus primers failed to detect viral RNA. One tissue sample (HKU66-Liver2) tested positive by the commercial RT-qPCR kit with a Cq value of 30.7 by one channel (FAM), but on further investigation was determined to be an off-target amplification.

### Serological investigation uncovers previous infection from multiple regions of origin

**Fig. 2:**
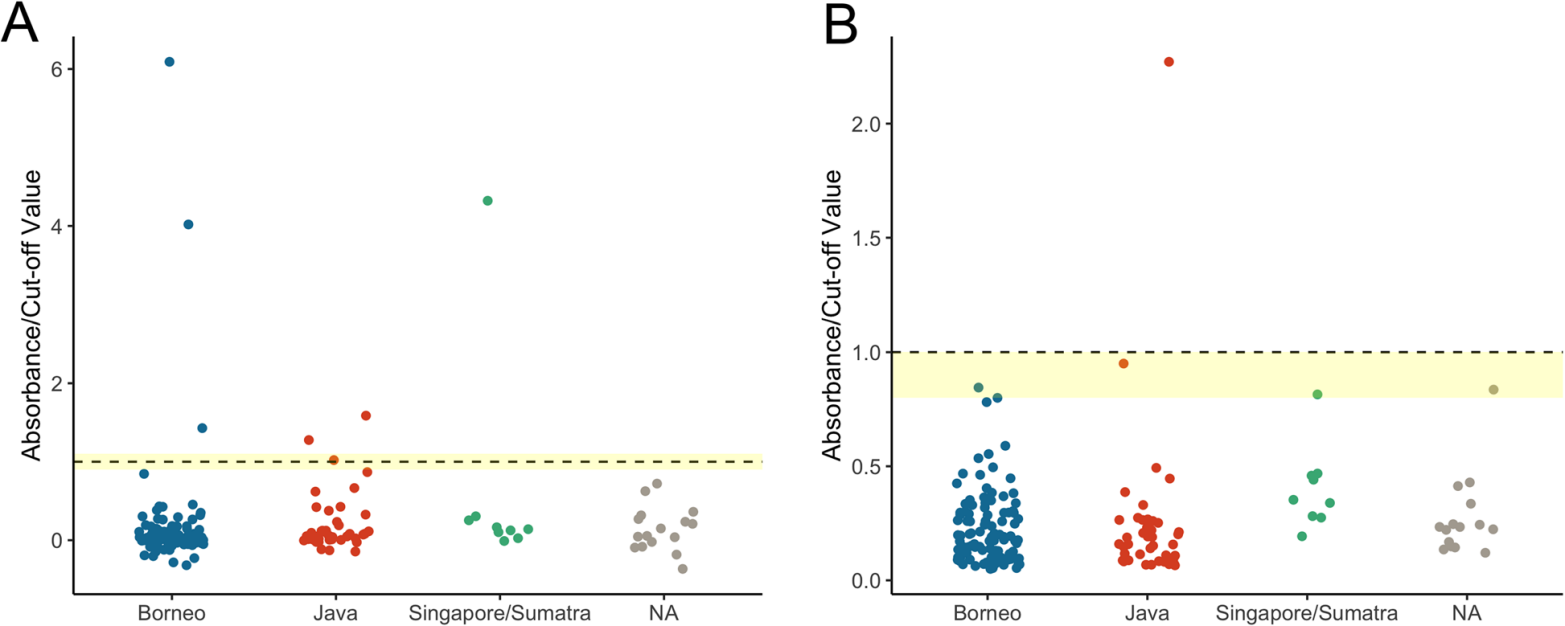
Serology results for 171 samples from 86 individuals, with all samples tested by the **A** WANTAI SARS-CoV-2 Ab ELISA (Wantai) targeting antibodies cross-reactive with the spike protein RBD, presenting the mean values for all samples tested in duplicate (except HKU64-PF), and for the **B** Platelia SARS-CoV Total Ab ELISA (Bio-Rad), targeting antibodies cross-reactive with the nucleocapsid protein (Additional file 3 for raw data from both assays). Dotted line represents seropositivity threshold of absorbance/cut-off value (A/CO) = 1.0 for both assays, while yellow shading indicates borderline seropositivity of 0.9 ~ 1.1 (inclusive) for the Wantai ELISA and represents an equivocal interpretation of 0.8 ~ 1.0 (inclusive) for the Bio-Rad ELISA. One sample was interpreted as seropositive by both assays, while six samples (representing five individuals) were interpreted as putatively seropositive (seropositive by the Wantai ELISA but equivocal or negative by the Bio-Rad ELISA). An additional eight samples were interpreted as seronegative but inconclusive with borderline or approaching-borderline results by the Wantai ELISA and/or equivocal results by the Bio-Rad ELISA (see Table 1)

Blood and various body fluids (*n* = 171) collected from 86 carcasses (58 from Borneo, 16 from Java, 8 from Singapore/Sumatra, and 4 of undetermined origin) were examined for presence of total antibodies cross-reactive with the SARS-CoV-2 spike protein receptor-binding domain (RBD) and nucleocapsid protein using a pair of double-antigen sandwich ELISAs. Sufficient materials for serological testing could not be retrieved for three carcasses and were thus omitted (HKU10, HKU53, and HKU54). Individuals from all regions, including Borneo, Java, and Singapore/Sumatra, were found to be seropositive or putatively seropositive (Table 1, Fig. 2). One individual from Java (HKU74) was found to be seropositive with support from both assays targeting the RBD and nucleocapsid protein. Two individuals from Java (HKU49 and HKU58) were putatively seropositive with support from only one assay, while two individuals (HKU05 and HKU55) did not meet the seropositivity threshold but was interpreted as inconclusive. Two individuals from Borneo (HKU66 and HKU37) were putatively seropositive, while two individuals (HKU29 and HKU51) did not meet the seropositivity threshold but were interpreted as inconclusive. One individual from Singapore/Sumatra (HKU75) was found to be putatively seropositive. One individual of undetermined origin (HKU15), putatively originating from Java based on mitogenome investigation (see mitogenome sequencing results below and Additional file 3), did not meet the seropositivity threshold but was considered inconclusive following a more lenient interpretation given the potential for infection by diverse SARSr-CoVs in pangolins and the reasonable expectation that this may result in reduced cross-reactivity with SARS-CoV-2 antigens. Concordance between the two ELISA methods was calculated to be 91.8% (157/171), with samples interpreted as inconclusive or as positive by only one ELISA method considered non-concordant.

**Table 1:** Summary of positive and inconclusive serological findings with overall interpretation based on congruency between both spike protein RBD and nucleocapsid protein ELISA results. Interpretations provided include “Positive (RBD & N)” if both the spike protein receptor binding domain (RBD) ELISA (Wantai) and nucleocapsid (N) protein ELISA (Bio-Rad) results are interpreted as positive; “Positive (RBD)” if the Wantai ELISA results are interpreted as positive but the Bio-Rad ELISA results are equivocal or negative; and “Inconclusive” if the results of either ELISA are interpreted as borderline, approaching-borderline, or equivocal without congruence between the two methods. Interpretation of individual ELISA results (see Additional file 3 for raw data from all samples with interpretations) was as follows: “***” indicates positive absorbance/cut-off value (A/CO) > 1.1 for the Wantai ELISA or positive ratio > 1.0 for the Bio-Rad ELISA; “**” indicates borderline value of 0.9 ~ 1.1 for the Wantai ELISA or equivocal ratio of 0.8 ~ 1.0 for the Bio-Rad ELISA; and “*” indicates a more lenient approaching-borderline value of 0.8 ~ 0.9 for the Wantai ELISA

Individual	Origin	Sample type	Spike protein RBD ELISA A/CO		Nucleocapsid protein ELISA A/CO	Interpretation
			**Replicate 1**	**Replicate 2**		
HKU05	Java	Organ fluid (OF)	0.108	0.124	0.950**	Inconclusive
HKU15	Undetermined	Organ fluid (OF)	0.629	0.813*	0.246	Inconclusive
		Pericardial fluid (PF)	0.558	0.692	0.836**	Inconclusive
HKU29	Borneo	Organ fluid (OF)	0.098	0.252	0.800**	Inconclusive
HKU37	Borneo	Organ fluid (OF)	1.937***	6.101***	0.845**	Positive (RBD)
HKU49	Java	Carcass fluid (CF)	1.012**	2.163***	0.159	Positive (RBD)
		Heart blood (HB)	0.919**	0.411	0.267	Inconclusive
HKU51	Borneo	Organ fluid (OF)	0.739	0.952**	0.350	Inconclusive
HKU55	Java	Carcass fluid (CF)	0.183	1.056**	0.493	Inconclusive
HKU58	Java	Carcass fluid (CF)	0.559	1.994***	0.230	Positive (RBD)
HKU66	Borneo	Organ fluid (OF)	3.247***	8.937***	0.462	Positive (RBD)
		Pericardial fluid (PF)	0.222	2.635***	0.425	Positive (RBD)
HKU74	Java	Carcass fluid (CF)−2	0.876*	0.860*	0.387	Inconclusive
		Heart blood (HB)	1.249***	0.793	2.271***	Positive (RBD & N)
HKU75	Singapore/Sumatra	Organ fluid (OF)	1.468***	7.174***	0.815**	Positive (RBD)

The testing of a subset of pangolin body fluid samples using the multiplex surrogate virus neutralization test (M-sVNT) against a panel of 12 different sarbecoviruses indicated that, of the subset of samples tested, none of those samples which were interpreted as putatively seropositive (HKU66-OF and HKU75-OF) or inconclusive (HKU74-CF-2) by the two ELISA methods could be confirmed positive by M-sVNT. Interestingly, organ fluid samples from HKU03 and HKU07 demonstrated a high level of inhibition for all sarbecoviruses included in the panel (Additional file 4). The presence of apparently broadly neutralizing antibodies in these two individuals could indicate a previous infection with a sarbecovirus, but these results cannot be readily explained as these samples did not contain antibodies which were cross-reactive with the SARS-CoV-2 spike RBD or nucleocapsid protein antigens utilized in the ELISAs. Failure to detect neutralizing antibodies by M-sVNT in samples which had tested putatively seropositive by means of one of the two ELISA methods may suggest exposure of the pangolins to more distantly related sarbecoviruses for which cross-neutralizing antibodies were not developed for the panel of sarbecoviruses included in the M-sVNT.

### Mitogenome sequencing demonstrates organ-carcass associations grouped by region

As some organs were not physically associated with their host carcasses in the seized samples, mitochondrial genome sequences were generated specifically for the purpose of associating loose organ samples with their carcass of origin. Some putatively seropositive samples were organ fluids retrieved from bags containing multiple sets of organs. To confirm the geographical origins of the serological samples, mitogenomes were obtained from the carcasses themselves as well as loose or bagged organs contained within the abdominal cavity. Investigation of mitogenomes from carcasses and associated organs revealed evidence for the presence of up to 120 Sunda pangolin individuals, but not including fetuses (*n* = 12) in the count of individuals (the count of unique mitogenomes obtained for each carcass and associated organs are provided in Additional file 3).

Mitogenomes obtained from the pectoral muscles of several carcasses were identical, indicating the presence of multiple closely related individuals in a single seizure (e.g., mother and offspring). While loose organ tissues obtained from within a carcass may be identical or share high identity with other carcasses, the comparison of mitogenomes alone is not sufficient to determine the precise individual from which these mixed organ tissues originated. Where multiple unique mitogenomes were obtained from mixed loose organ tissues, there was an apparent grouping of mitogenomes by region, with the exceptions being HKU02 and HKU13. For example, a mitogenome obtained from loose organs inside a Borneo-origin carcass, while distinct from the mitogenome obtained from that individual’s carcass, was still most similar to other mitogenomes obtained from Borneo-origin carcasses. This suggests that the geographic origins estimated for the putatively seropositive organ fluids would not be confounded by the organ assortment. In the case of HKU02 and HKU13 (both seronegative), variation in the mitogenomes obtained from the loose organs in these carcasses prevented a clear association with mitogenomes from any one region and thus the putative origin remains undetermined.

## Discussion

Our results integrate conservation genetics, virological, and serological methods to contextualize the origins and potential pathogen exposures of trafficked pangolins within a complex trade network. Our population genetic analysis indicated that the pangolins from these seizures in 2013 and 2018 can be assigned to three different origins within the Sunda pangolin range, suggesting that Hong Kong is an important node in the trade network, consistent with other studies [21, 31]. Furthermore, multiple pangolin origins found in a single Hong Kong seizure (in 2018) points to organized and transnational efforts behind the trade of Sunda pangolins from Southeast Asia. As opposed to being traded along local and independent trade routes, Sunda pangolin products are likely separately sourced from their wild habitats then transported as a group across the region. This further supports the involvement of criminal syndicates in the trafficking of Sunda pangolins in Asia, which is known in Sumatra [40], and that there is active cross-border cooperation between traffickers across Southeast Asia, which has also been found for other regions and Asian pangolin species (e.g., Aditya et al. [41] in India; Archer et al. [42] for the Philippine pangolin). As the trade continues to persist despite CITES listing of all pangolin species, the existence of various protective legislation across the region, and speculations that pangolins might serve as an intermediate host of coronaviruses, this scale of trade calls for urgent and decisive conservation attention across institutional and geographical scales.

While we were unable to detect viral RNA by means of PCR for coronaviruses or paramyxoviruses, we found serological evidence for previous SARSr-CoV infection in a pangolin originating from Java and putatively seropositive individuals from all three geographic regions. However, the application of serological methods for diverse wildlife species faces challenges for appropriate validation, especially in the absence of detection of viral RNA. This is further restricted by a paucity of information regarding pangolin immunology and the duration of antibody persistence following SARSr-CoV infections, although studies have identified similarities between SARS-CoV-2 infection in humans and SARSr-CoV infection in Sunda pangolins [3, 9]. It is unclear how long antibodies will persist in pangolins following infection with SARSr-CoVs, although in the case of human infection by SARS-CoV and SARS-CoV-2, antibodies appear to rapidly decay following recovery, followed by a gradual decline in the rate of decay until antibody titers become undetectable [43–45]. It cannot be excluded that pangolins included in this study may have recovered from past SARSr-CoV infection and could have diminished antibody titers, failing to meet the seropositivity thresholds or undetectable by means of the ELISAs. The double-antigen sandwich assay format employed by the two ELISA kits used in this study has been previously used for pangolins infected with SARSr-CoVs [3]. The inconsistency observed between ELISA and M-sVNT results obtained for HKU03 and HKU07 organ fluid samples, both of which were broadly neutralizing in M-sVNT but which did not test positive by either ELISA kit, cannot be readily explained except that some undetermined molecule present in the organ fluids may be interfering with the M-sVNT assay. This M-sVNT assay has not been validated for pangolins, and thus there is potential for unpredicted interference. High concordance was observed between the spike RBD ELISA (Wantai) and N protein ELISA (Bio-Rad), and while these results are not further supported in the subset of samples also tested by M-sVNT, it is not incompatible that samples which test putatively seropositive by ELISA may not exhibit neutralizing activity against the panel of specific sarbecoviruses included in the M-sVNT.

**Fig. 3:**
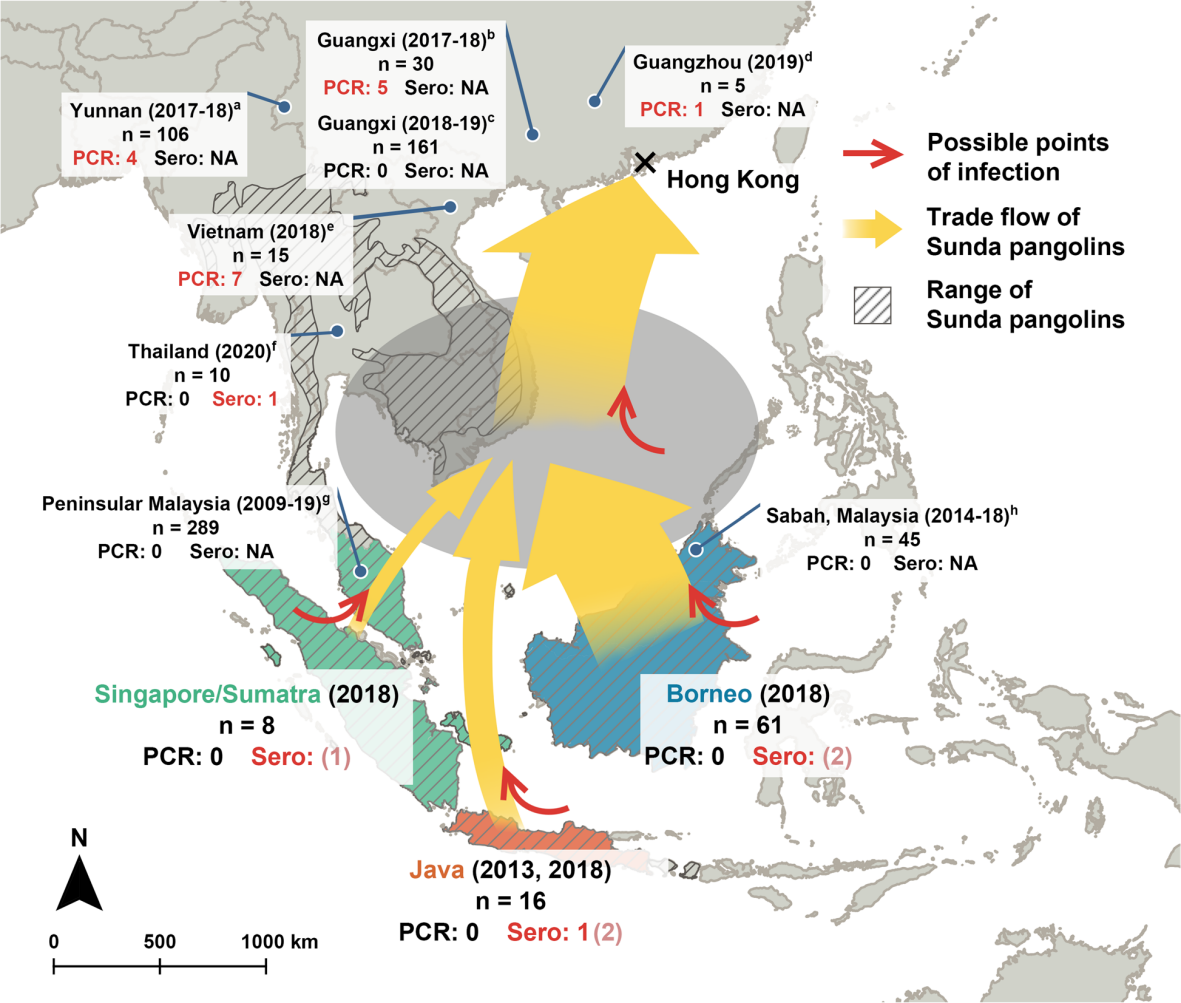
Summarized serological and population genomics results show complex relationships between Sunda pangolin trade and origins of pangolin SARSr-CoVs in Southeast Asia. The gray oval indicates unknown trade intermediaries where trafficked pangolins are processed. Red arrows indicate possible points where trafficked pangolins are infected with SARSr-CoVs, thereby entry points of SARSr-CoVs into the trade. Yellow arrows indicate the inferred trade flow of Sunda pangolins analyzed in this study and are proportional to number of individuals found from each population within the seizure under study. Sunda pangolin range data sourced from IUCN [16]. Blue points show results of published literature testing for SARSr-CoVs in pangolin individuals (*n* = total number of individuals tested), with PCR and serological results separated, wherein positive results are in red and positive (inconclusive) results are in light pink. Years in brackets indicate seizure year. Results from the literature are attributed geographically to seizure locations, while results from this study are shown according to pangolin origins (Singapore/Sumatra, Java, and Borneo). Studies used in this figure are numbered as follows: (a) Peng et al. [12]; (b) Lam et al. [1]; (c) Shi et al. [37]; (d) Lam et al. [1]; (e) Nga et al. [11]; (f) Wacharapluesadee et al. [13]; (g) Lee et al. [49]; and (h) Lee et al. [49]

The pangolin samples with putative serological evidence of past SARSr-CoV infection originate from all three geographic regions, indicating that there may be complex underlying interactions between the trade and associated pathogens (Fig. 3). Given that these pangolins are likely held at an intermediate location between the source and their destination, our findings are not sufficient to determine whether these pangolins were infected with SARSr-CoVs in their natural habitat or through the course of trafficking. Pangolins have been suspected to be maintenance hosts for zoonotic diseases due to their observed associations with ticks as well as other diverse parasites and pathogens [34–37, 46, 47], although the harsh conditions that traded pangolins are put in confounds whether specific associations occur naturally or through trade [48]. The existing literature indicates that SARSr-CoVs in pangolins are mostly detected at relatively downstream points of seizure [1–3, 11, 12], while pangolins seized upstream have not been discovered to harbor these viruses [49]. However, most studies thus far have not included a serological investigation of trafficked pangolins which is problematic because PCR negative samples are not necessarily evidence of the absence of coronaviruses within smuggled carcasses given the potential for RNA degradation. Serological techniques are useful to uncover broader patterns of infection prevalence and past exposure. It is evident that comprehensive screening and monitoring of pathogens in trafficked wildlife, especially more expansive serological studies, are needed to effectively identify emerging pathogens with zoonotic potential and to better understand the host breadth of these pathogens.

## Conclusions

Considerable attention has been directed towards detection of SARSr-CoVs in wildlife that are traded and/or consumed to better understand the diversity of SARSr-CoVs in wildlife posing zoonotic risk, and in the investigation into the origins of COVID-19. However, a litany of gaps remains. Perhaps one of the more significant is that there is a lack of understanding of the relationship between potentially zoonotic pathogens and traded wildlife [50], which can be attributed to the lack of effort to contextualize the wildlife in terms of their trade routes and provenance. Our integrated results highlight the need to understand the complexity of wildlife trade and how that affects pathogen dynamics across geographic and taxonomic scales to improve future surveillance efforts and understanding of wildlife-pathogen interactions in trade.

## Methods

### Necropsy examination and sample collection

Carcasses were seized in 2013 (*n* = 1) and in 2018 (*n* = 88) as part of anti-smuggling operations conducted within Hong Kong SAR through coordinated efforts of Hong Kong Customs and Excise Department, the Hong Kong Police Force, and the Agriculture, Fisheries and Conservation Department (AFCD) of The Government of the Hong Kong SAR. Seized carcasses, frozen and partially dressed for human consumption at the time of confiscation, had been kept frozen at − 20 °C and were donated for use in this study in May 2020 in coordination with AFCD and Kadoorie Farm and Botanic Garden (KFBG).

Post-mortem examination was conducted at Ocean Park Corporation for each animal following a standard necropsy procedure after thawing at 4 °C. Oropharyngeal, nasal, and rectal swabs were collected and stored in virus transport media (VTM). Throughout the post-mortem examination, tissue samples were collected from all major organ systems using standard sterile techniques in microbiological practice and stored in VTM or phosphate-buffered saline (PBS). Samples were transported by cold chain to the State Key Laboratory of Emerging Infectious Diseases, The University of Hong Kong, where further sample processing was conducted.

### Population genomics

As origins of all 89 individuals in the seizure were unknown, we used a population genomics approach similar to that in Tinsman et al. [51] to assign all individuals to three wild populations within the species previously identified by Nash et al. [32] and Sitam et al. [38] using ddRADseq. By including wild individuals sequenced by Nash et al. [32, 52], the seized individuals can be grouped based on SNPs, wherein the population origin of each group can be identified by the wild individuals of known origin present in the group.

#### DNA extraction and ddRADseq library preparation

DNA was extracted from pectoral muscle tissue of each of the 89 carcasses using the DNeasy Blood and Tissue Kit (Qiagen). Library preparation for ddRADseq was performed following Nash et al. [32] and Peterson et al. [53], using EcoRI and MspI as the restriction enzymes. Libraries were divided into two pools and selected for a size range of 250–600 bp with a BluePippin (Sage Science), then finally sequenced using the Illumina NovaSeq 6000 system at the Centre of PanorOmic Sciences (CPOS), The University of Hong Kong.

#### Bioinformatic analyses

Raw sequencing reads were checked with FastQC v0.11.9 [54] then demultiplexed and truncated with process_radtags in STACKS v2.54 [55]. Truncated reads were aligned to a reference Sunda pangolin genome [56, 57] with bwa_memscript [58]. Sequence data of wild individuals (i.e., references) from Java (*n* = 3), Borneo (*n* = 2), and Singapore/Sumatra (*n* = 7) provided by Nash et al. [32, 52] were also included in the sample pool as references. SNPs were called with ref_map.pl in STACKS v2.54 [55]; loci were discarded if they did not occur in at least 95% of individuals (-R 0.95), and only the first SNP from each locus was kept (–write-single-snp). SNPs were then subject to further quality checks with PLINK 1.9 [59], filtering out individuals with more than 10% missing data (–mind), and filtering out SNPs with minor allele frequency below 0.01 (–maf) and *p* value of Hardy–Weinberg equilibrium test below 0.01 (–hwe). Loci were further checked for linkage disequilibrium (–indep-pairwise) and checked for neutrality with BayeScan v2.1 with 10,000 iterations [60].

To assign individuals to populations, PCA was first conducted with the SNPRelate package in R as a preliminary assessment [61, 62]. We then conducted a STRUCTURE analysis for a range of one to five genetic clusters (*K*) with the admixture model [63], with five repetitions of 50,000 Markov chain Monte Carlo (MCMC) iterations and a 10,000 burn-in period for each *K* value. Optimal *K* value was determined by delta-*K* according to the Evanno method implemented in the pophelper package in R [64]. Finally, we also conducted a fineRADstructure analysis to further inform population substructure and possible admixture [65], following the recommended settings.

### Detection of viral RNA

#### Sample processing and nucleic acid extraction

Swab samples were briefly vortexed then centrifuged for 2 min at 12,000 rpm, and 140 µl supernatant was used as input for extraction of RNA using the QIAamp® Viral RNA Mini Kit (Qiagen) following manufacturer’s instructions without addition of carrier RNA and with a double elution step using 30 µl Buffer AVE for each elution following a 5 min on-column incubation at room temperature and stored at − 80 °C. Extracted RNA was used in downstream complementary DNA (cDNA) synthesis and for detection of target viruses by PCR methods.

For tissue samples collected during post-mortem examination, RNA was extracted using the RNeasy® Plus Micro Kit (Qiagen). Buffer RLT Plus was prepared according to the manufacturer’s instructions without addition of carrier RNA. Approximately 30 mg of organ tissue with an equal quantity of RNase-free stainless-steel beads were homogenized in a Bullet Blender® Blue (Chembio Ltd.) tissue homogenizer and used as input for extraction. Elution was performed twice, using 30 µl RNase-free double-distilled water (ddH_2_O) and following a 5 min on-column incubation at room temperature. Extracted RNA was used for downstream PCR detection of target viruses.

#### Synthesis of complementary DNA

cDNA was synthesized from the extracted RNA using PrimeScript™ II 1st strand cDNA Synthesis Kit (TaKaRa Bio). Synthesis of cDNA was performed following the manufacturer’s instructions. The prepared cDNA was then used for diagnostic screening by conventional PCR.

#### Coronavirus detection

Diagnostic screening for coronaviruses was performed by RT-PCR for broad detection of coronaviruses and by RT-qPCR for specific detection of sarbecoviruses and for the detection of SARS-CoV-2 specifically using a commercial kit.

Universal detection for members of the family *Coronaviridae* was performed using RT-PCR targeting a conserved region of the RNA-dependent RNA polymerase (RdRp) gene region, producing an amplicon of approximately 442 base-pairs (bp) [66] and following a thermocycling protocol as previously described by Miot et al. [67].

Consensus primers for members of the genus *Sarbecovirus* were designed for RT-qPCR to re-test the Sunda pangolin swab and tissue samples as a more sensitive approach than the pan-coronavirus RT-PCR but with less specificity than a commercial reverse transcription-quantitative PCR (RT-qPCR) kit. Two-step RT-qPCR was performed using TB Green® Premix Ex Taq™ (Tli RNase H Plus) (TaKaRa Bio), with cDNA used as input following the reverse transcription of RNA as described above. The reaction contained 10 µl TB Green Premix Ex Taq (Tli RNase H Plus) (2X), 0.4 µl forward primer SarbeCoV-F (5′-GTTAAGAGRCAYACKWTSTCTAACTA-3′) (10 mM), 0.4 µl reverse primer SarbeCoV-R (5′-CGTGATATATGTGGTACCATGTCACC-3′) (10 mM), 1 µl of cDNA template (or 2 µl of pooled cDNA template at 0.4 µl per sample), topped up to 20 µl reaction volume with ddH_2_O. Thermocycling was performed with an initial denaturation of 95 °C for 30 s, followed by 40 cycles of 95 °C for 5 s and 60 °C for 20 s, with a final melt curve analysis of 95 °C for 0 s, 65 °C for 15 s, and 95 °C for 0 s.

RT-qPCR was performed for the Sunda pangolin swab and tissue samples using the commercial Hybribio COVID-19 Real-Time PCR kit (Chaozhou Hybribio Biochemistry Ltd), with high specificity for SARS-CoV-2. This assay used multiple fluorescence channels for detection of the SARS-CoV-2 open reading frame 1ab (ORF1ab) and nucleoprotein coding sequence (N CDS), as well as the β2-microglobulin gene for an internal control. The reaction was performed according to the manufacturer’s instructions.

#### Paramyxovirus detection

Diagnostic screening for paramyxoviruses (family *Paramyxoviridae*) was performed using a semi-nested RT-PCR targeting the polymerase pol gene region [68] and using a modified thermocycling protocol as described by Lee et al. [49].

### Serological investigation

Hemolyzed body fluids, blood, urine, and amniotic fluid were collected during post-mortem examination of seized pangolin carcasses. Fluids collected from 86 individuals were examined for presence of total antibodies reactive or cross-reactive with the RBD of the SARS-CoV-2 spike protein and SARS-CoV-2 nucleocapsid protein. Detection of anti-SARS-CoV-2 spike RBD antibodies and anti-SARS-CoV-2 nucleocapsid antibodies was investigated by means of a two-step and one-step double-antigen sandwich ELISA respectively, techniques demonstrated to successfully detect total SARSr-CoV antibodies in diverse species including pangolins [3, 67, 69]. The high similarity between the viral proteins characterized from pangolin SARSr-CoVs and SARS-CoV-2 justifies the use of this assay in the detection of antibodies cross-reactive with diverse SARSr-CoV antigens. Previous work examining total antibodies against SARS-CoV-2 in Sunda pangolins demonstrated the efficacy of these types of diagnostic assays for detection of SARSr-CoVs with cross-reactivity to the SARS-CoV-2 spike protein [3]. An evaluation of the ELISAs utilized in this work demonstrated reasonably high concordance (89.5%) between these methods as applied to human clinical samples, though discrepancies were observed, especially for samples collected from patients exhibiting limited COVID-19 symptoms [70]. Prior studies have demonstrated that similar ELISAs had limited cross-reactivity due to prior infection with other diverse alpha- and betacoronaviruses [67, 71, 72], with strongest cross-reactivity observed between the SARS-CoV-2 spike protein and SARS-CoV or Middle East respiratory syndrome coronavirus (MERS-CoV) antibodies [71].

Whole blood and fluid samples were centrifuged at 2000 rpm for 10 min in a centrifuge pre-cooled to 4 °C to separate cellular fractions from whole blood and to remove sediment from other fluid samples, with the hemolyzed serum or supernatant used in serological assays. Retrieval of non-hemolyzed serum was not possible due to the frozen storage of these carcasses prior to our investigation taking place. Serological investigation of post-mortem samples have been demonstrated to be equally as effective as ante-mortem samples for infectious disease screening [73]. While the persistence of antibodies post-mortem is impacted by environmental conditions, especially temperature, detection of antibodies in decomposing carcasses has been successfully utilized in wildlife disease outbreak investigations [74].

Detection of antibodies reactive against SARSr-CoVs in carcass and organ fluids collected from Sunda pangolins was performed following sample preparation and removal of sediment. The WANTAI SARS-CoV-2 Ab ELISA Diagnostic Kit (Beijing Wantai Biological Pharmacy Enterprise Co. Ltd), utilizing the double-antigen sandwich ELISA format, was used for detection of total antibodies with activity against the SARS-CoV-2 spike protein RBD. The Platelia SARS-CoV-2 Total Ab (Bio-Rad Laboratories, Inc.) ELISA, utilizing a one-step double-antigen sandwich ELISA, was used for detection of total antibodies with activity against the SARS-CoV-2 nucleoprotein. In principle, these diagnostic tests should work broadly with blood and fluid samples collected from diverse host species as no species-specific secondary antibody is used during the protocol.

Absorbance was measured using a FilterMax™ F5 Multi-Mode Microplate Reader (Molecular Devices) using the dual wavelength setting with reference wavelength set to 620 nm and absorbance measured at 450 nm. All samples were tested in duplicate for the WANTAI SARS-CoV-2 Ab ELISA according to the manufacturer’s instructions, excepting one sample (HKU64-PF) for which the volume was insufficient, and cut-off values for each replicate were calculated with positive values having an absorbance/cut-off value (A/CO) > 1.1, negative values having an A/CO < 0.8, borderline values interpreted as having an A/CO in the 0.9 ~ 1.1 range, and approaching-borderline values leniently interpreted as having an A/CO in the 0.8 ~ 0.9 range. All samples were tested once by the Platelia SARS-CoV-2 Total Ab ELISA and cut-off values, or specimen ratios, were calculated with positive values having an A/CO > 1.0, negative values having an A/CO < 0.8, and equivocal values interpreted as having an A/CO in the 0.8 ~ 1.0 range.

For the Wantai ELISA, samples which yielded a positive result in one or both replicates were interpreted as positive, samples which yielded a borderline result in both replicates were interpreted as borderline, samples which yielded a borderline result in only one replicate were interpreted as borderline but inconclusive, samples which yielded an approaching-borderline result in only one replicate were interpreted as approaching-borderline, and samples which yielded a negative result in both replicates were interpreted as negative. For the Platelia ELISA, samples which yielded a positive result were interpreted as positive, samples which yielded a negative result were interpreted as negative, and samples with an equivocal result were interpreted as inconclusive. The Platelia ELISA was used to supplement the interpretation of the Wantai ELISA, with congruency between the two methods being treated as confirmation, and incongruency suggesting an inconclusive interpretation of the Wantai ELISA.

Body fluids were also tested using a M-sVNT platform as previously described [75, 76]. Briefly, the blood and body fluid samples were pre-incubated with RBD-coated MagPlex-Avidin Microspheres (Luminex) for 1 h at 37 °C with agitation, followed by addition of PE-labeled human angiotensin-converting enzyme 2 (ACE2) at a final concentration of 1000 ng/ml for an additional 30 min at 37 °C. After two PBS washes, the signals were acquired using a MAGPIX Instrument (Luminex). RBDs from 12 betacoronavirus strains were used in the M-sVNT assay, including human SARS-CoV-2 Wuhan wild type, Alpha, Beta, Delta, Gamma variants, human SARS-CoV, WIV-1, LYRa11, RsSHC014, Rs2018B, RaTG13, and GX-P5L.

### Mitogenome sequencing and assembly

Recovery of mitochondrial genome sequences derived from the pangolin carcasses themselves, as well as from detached organ tissues held in plastic bags within the abdominal cavity of carcasses, was performed to determine congruence between individual carcasses and the sets of loose organs. If the contents of the organ bag contained a single set of organs, then a piece of tissue from a single organ (lung or liver tissue where present) was used for mitogenome sequencing. If multiple sets of organs from multiple individuals were found (e.g., multiple sets of kidneys, livers, lungs, or spleens), mitogenome sequencing was conducted for each of these organs to identify the likely carcass of origin if present among the 89 individuals examined.

The full mitochondrial genome was amplified by three overlapping long PCRs (primers listed in Table S1). Long PCR was carried out in 20 µl reaction mixtures containing 4 µl 5X GXL Buffer, 1.6 µl of deoxynucleotide triphosphate (dNTP) mixture (2.5 mM each), 0.4 µl of each forward and reverse primer (10 mM each), 0.4 µl of GXL DNA polymerase (1.25 U/µl) (TaKaRa), 12 µl of ddH_2_O, and 1.2 µl of extracted DNA template obtained using the DNeasy Blood and Tissue Kit (Qiagen). The PCR conditions were denaturation at 98 °C for 2 min; 30 cycles of 98 °C for 10 s, 55 °C for 15 s, 68 °C for 7 min; and final extension at 68 °C for 10 min. Successful amplicons were screened on 1% agarose gel and further purified using the Expin PCR SV Kit (GeneAll) before proceeding to KAPA HyperPrep Library Preparation Kit (Roche), according to manufacturer instructions with adaptation to Adapterama system [77]. Libraries were subsequently sequenced using the Illumina NovaSeq 6000 system at CPOS for 2 × 150 bp paired-end reads.

Raw next-generation sequencing (NGS) reads were processed by fastp v0.20.1 [78] to conduct quality and adapter trimming as well as mitogenome primers removal. Trimmed reads were used to generate consensus sequences with Snippy v4.6.0 (https://github.com/tseemann/snippy), including reference-based mapping, variant calling, and consensus generation. The National Center for Biotechnology Information (NCBI) GenBank mitogenome sequences of *Manis javanica* [79] was used as reference. The resulting consensus mitogenome sequences were aligned back with the corresponding trimmed reads using minimap2 v2.22 [80] and manually checked with Integrative Genomics Viewer (IGV) 2.10.3 [81].

Manual editing of mitogenome sequences was performed using Geneious Prime® 2020.1.2 (Biomatters Ltd.) and multiple sequence alignment was performed using multiple alignment using fast Fourier transform (MAFFT) v7.450 [82] as implemented in Geneious Prime. Mitogenomes obtained from carcass pectoral muscle and loose organ tissues associated with an individual carcass were considered to be from different individuals if they contained > 3 nucleotide differences, allowing for minor variation within an individual.

## Supplementary Information

Additional file 1: Principal component analysis results of SNPs from ddRADseq. Analysis is based on 23,261 SNPs from 93 individuals (85 samples and 8 references), showing three distinct genetic clusters

Additional file 2: List of samples used for population genetics analysis, including seized carcasses and wild references from ChengNash et al. [31]. Samples not included in the final analysis were all filtered out by PLINK for missing data. Origins of seized samples inferred from PCA, STRUCTURE, and fineRADstructure analysis, while origins of wild references are taken from ChengNash et al. [31]

Additional file 3: Summary of serological findings with origins and number of unique mitogenomes obtained for all individuals. Interpretation of ELISA results as follows:“***” indicates positive >1.1 for spike protein RBD ELISA (Wantai) or positive >1.0 for nucleocapsid protein ELISA (Bio-Rad); “**” indicates borderline 0.9~1.1 for the Wantai ELISA or equivocal 0.8~1.0 for the Bio-Rad ELISA; and “*” indicates approaching-borderline 0.8~0.9 for the Wantai ELISA.“^” indicates organ fluid from HKU01, HKU03, and HKU07 which tested positive for antibodies cross-reactive with SARSr-CoVs by M-sVNT

Additional file 4: M-sVNT data for percent inhibition from the subset of individuals investigated. Abbreviations for sample types include organ fluid (OF), amniotic fluid (AmF), and carcass fluid (CF). “^” indicates organ fluid from HKU03 and HKU07 which tested positive for antibodies cross-reactive with SARSr-CoVs by M-sVNT. “***” indicates HKU66-OF sample which tested positive by spike protein RBD ELISA (Wantai) and negative by nucleocapsid protein ELISA (Bio-Rad). “**” indicates HKU75-OF sample which tested positive by the Wantai ELISA and equivocal by the Bio-Rad ELISA. “*” indicates HKU74-CF-2 sample which tested approaching-borderline by the Wantai ELISA and negative by the Bio-Rad ELISA

Additional file 5: Primer information for mitogenome sequencing. Oligonucleotide primer sequences used for amplification of overlapping fragments of the pangolin mitogenomes

## Data Availability

All supplementary data associated with the paper are available from the online web link. Sequencing data acquired through ddRADseq and the Sunda pangolin mitogenome data have been deposited in the NCBI SRA database under BioProject PRJNA954554 [52].

## Abbreviations

ACE2: Angiotensin-converting enzyme 2
A/CO: Absorbance/cut-off value
AFCD: Agriculture, Fisheries and Conservation Department of Hong Kong
AmF: Amniotic fluid
cDNA: Complementary DNA
CDS: Coding sequence
CF: Carcass fluid
CoV: Coronavirus
COVID-19: Coronavirus disease 2019
CPOS: Centre of PanorOmic Sciences, The University of Hong Kong
ddH_2_O: Double-distilled water
ddRADseq: Double digest restriction site-associated DNA sequencing
DNA: Deoxyribonucleic acid
dNTP: Deoxynucleotide triphosphate
ELISA: Enzyme-linked immunosorbent assay
HB: Heart blood
HKU: The University of Hong Kong
IGV: Integrative Genomics Viewer
KFBG: Kadoorie Farm and Botanic Garden
MAFFT: Multiple alignment using fast Fourier transform
MCMC: Markov chain Monte Carlo
MERS-CoV: Middle East respiratory syndrome coronavirus
M-sVNT: Multiplex surrogate virus neutralization test
N: Nucleocapsid protein
NCBI: National Center for Biotechnology Information
NGS: Next-generation sequencing
OF: Organ fluid
ORF1ab: Open reading frame 1ab
PBS: Phosphate-buffered saline
PCA: Principal component analysis
PCR: Polymerase chain reaction
PF: Pericardial fluid
RBD: Receptor-binding domain
RdRp: RNA-dependent RNA polymerase
RNA: Ribonucleic acid
RT-PCR: Reverse transcription PCR
RT-qPCR: Reverse transcription-quantitative PCR
SARS: Severe acute respiratory syndrome
SARS-CoV: Severe acute respiratory syndrome coronavirus
SARS-CoV-2: Severe acute respiratory syndrome coronavirus 2
SARSr-CoV: SARS-related coronavirus
SNPs: Single nucleotide polymorphisms
VTM: Virus transport media
