# Supplementary material for: Serological evidence of sarbecovirus exposure along Sunda pangolin trafficking pathways
Source: BMC Biol. 2024 Nov 26;22:274. doi: 10.1186/s12915-024-02074-x (PMC11600613; doi:10.1186/s12915-024-02074-x)
Supplement: Supplementary file 5 — Additional file 5: Primer information for mitogenome sequencing. Oligonucleotide primer sequences used for amplification of overlapping fragments of the pangolin mitogenomes [file 12915_2024_2074_MOESM5_ESM.docx]

**Table S4.** Primer information for mitogenome sequencing

PCR ID Primer Sequence (5' - 3')

1 Manis_Mito_336F CCACCGCGGTCATACGATTA

Manis_Mito_6030R TCGGGGTGTCCGAAGAATCAG

2 Manis_Mito_5615F GACATGGCATTCCCCCGTAT

Manis_Mito_12392R CGGATTTTCCAGTTGCTGCTAG

3 Manis_Mito_12240F GACGAACAGATGCAAACACAGC

Manis_Mito_490R GTGGGGTATCTAATCCCAGTTTG
